# Evaluation of the Diagnosis and Antibiotic Prescription Pattern in Patients Hospitalized with Urinary Tract Infections: Single-Center Study from a University-Affiliated Hospital

**DOI:** 10.3390/antibiotics12121689

**Published:** 2023-12-01

**Authors:** Adina Fésüs, Mária Matuz, Erika Papfalvi, Helga Hambalek, Roxána Ruzsa, Bence Tánczos, Ildikó Bácskay, István Lekli, Árpád Illés, Ria Benkő

**Affiliations:** 1 Department of Pharmacology, Faculty of Pharmacy, University of Debrecen, H-4032 Debrecen, Hungary; fesus.adina@pharm.unideb.hu (A.F.); tanczos.bence@med.unideb.hu (B.T.); lekli.istvan@pharm.unideb.hu (I.L.); 2 Department of Pharmaceutical Technology, Faculty of Pharmacy, University of Debrecen, H-4032 Debrecen, Hungary; bacskay.ildiko@pharm.unideb.hu; 3 Institute of Healthcare Industry, University of Debrecen, H-4032 Debrecen, Hungary; 4 Clinical Pharmacy Department, Faculty of Pharmacy, University of Szeged, H-6725 Szeged, Hungary; matuz.maria@szte.hu (M.M.); papfalvi.erika.piroska@med.u-szeged.hu (E.P.); hambalek.helga@szte.hu (H.H.); ruzsa.roxana@szte.hu (R.R.); 5 Central Pharmacy, Albert Szent Györgyi Medical Center, University of Szeged, H-6725 Szeged, Hungary; 6 Department of Emergency Medicine, Albert Szent Györgyi Medical Center, University of Szeged, H-6725 Szeged, Hungary; 7 Department of Internal Medicine, Faculty of Medicine, University of Debrecen, H-4032 Debrecen, Hungary; illes.arpad@med.unideb.hu

**Keywords:** urinary tract infections, empirical antibiotic therapy, guideline adherence, diagnosis, misdiagnosis

## Abstract

UTIs (urinary tract infections) are common bacterial infections with a non-negligible hospitalization rate. The diagnosis of UTIs remains a challenge for prescribers and a common source of misdiagnosis. This retrospective observational study aimed to evaluate whether recorded diagnosis by clinicians and empirical antibiotic therapy met the EAU (European Association of Urology) guideline in patients hospitalized with UTI. The study was conducted at an internal medicine unit of a tertiary care medical center in Hungary. The diagnosis was assessed based on clinical presentation, physical examination, and laboratory (including microbiological) results, considering all the potential risk factors. Diagnosis was considered misdiagnosis when not confirmed by clinical presentation or clinical signs and symptoms. Evaluation of empirical antibiotic therapy was performed only for confirmed UTIs. Empirical treatment was considered guideline-adherent when complying with the relevant recommendations. Out of 185 patients, 41.6% failed to meet EAU-based UTI diagnosis criteria, of which 27.6% were misdiagnosed and 14.1% were ABU (asymptomatic bacteriuria). The diagnosis of urosepsis recorded at admission (9.7%, 18/185) was not confirmed either by clinical or microbiological tests in five (5/18) cases. The initial empirical therapies for UTI showed a relatively low rate (45.4%) of guideline adherence regarding agent selection. The most common guideline-non-adherent therapies were combinations with metronidazole (16.7%). Dosage appropriateness assessments showed a guideline adherence rate of 36.1%, and underdosing due to high body weight was common (9.3%). Overall (agent, route of administration, dose, duration) guideline adherence was found to be substantially low (10.2%). We found a relatively high rate of misdiagnosed UTIs. Written protocols on the ward may be crucial in reducing misdiagnosis and in optimizing antibiotic use.

## 1. Introduction

Inappropriate use of antibiotics has led to ABR (antimicrobial resistance), which is now reckoned as the most severe global public health threat [1,2]. UTIs (urinary tract infections) are common bacterial infections, and are the fourth most common cause of death associated with ABR worldwide [3]. Although UTIs are often treated in ambulatory settings, the hospitalization rate is not negligible. The epicenter of ABR is attributable to the hospital environment [4,5]. A study conducted in the USA found that the hospitalization rate with UTIs was 15.5% [6], and these infections accounted for 2.3% of all deaths in hospitalized patients [7].

In acute care hospitals in Europe, about 35% (in Hungary it was 30%) of patients received at least one antimicrobial agent during their stay [8,9]. ECDC (European Centre for Disease Prevention and Control, Solna, Sweden) point prevalence survey data showed that antibiotic use for UTIs in Europe represents 16% of all antibacterial use in acute care hospitals [9]. CAIs (community-acquired infections) account for 69.9% of the total hospital infections treated with antibiotics, and 15% of these were found to be UTIs (Hungary: 71.8% and 14%, respectively) [10].

Since antibiotics play a key role in the treatment of UTIs, their misuse or overuse may lead to antibiotic resistance (ABR), posing severe health problems for patients and high costs for society [11]. Moreover, the empirical antibiotic therapy for UTIs is becoming a challenge for prescribers, since there are a number of available classifications of UTIs and various guidelines for empirical antibiotic treatment [12,13]. Nevertheless, the diagnosis of UTIs remains a common source of misdiagnosis [12,13]. UTIs are one of the most common overdiagnosed infections treated in hospitals [14], mainly in patients presenting at emergency departments with non-UTI complaints [15,16]. Moreover, misdiagnosis may lead to further unnecessary antibiotic exposure and delay in real diagnosis [17]. Furthermore, in Hungary (2017), in the absence of a national guideline for all UTI types, only international (EAU) guidelines could serve as a recommendation.

To date, descriptions of antibiotic treatment trends for UTIs have only been published for adult primary care in Hungary [18,19]. Despite the frequency and importance of UTIs, no field studies have been conducted in patients hospitalized in Hungary to evaluate the diagnosis and the first empirical antibiotic treatment(s).

The aim of this study was to evaluate the diagnosis and antibiotic prescription pattern for UTIs in hospitalized patients.

## 2. Results

**Figure 1:**
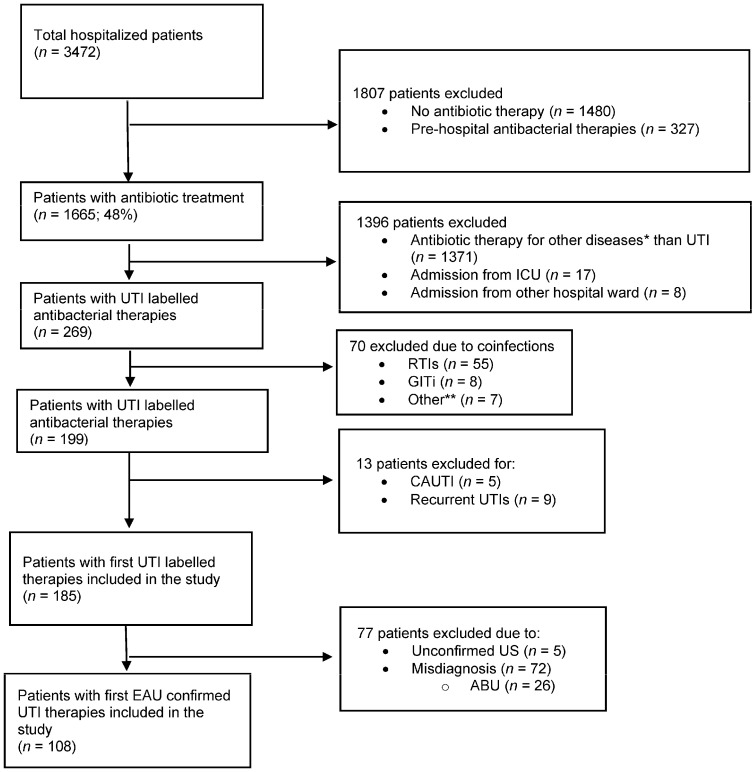
Flowchart for exclusion and inclusion criteria. * Other diseases including sepsis, surgical site infections, etc.; UTI: urinary tract infection; ICU: intensive care unit; RTIs: respiratory tract infections; GITi: gastrointestinal tract infections; **: other coinfections including decubitus, skin and soft tissue infections; CAUTI: catheter-associated urinary tract infection; US: urosepsis; ABU: asymptomatic bacteriuria.

During the study period, 1665 patients had data gathered; of those, 185 patients’ data were deemed suitable for inclusion in the analysis based on the study criteria. Figure 1 displays the inclusion and exclusion criteria for the research.

### 2.1. Patient Characteristics and Main Outcomes

The characteristics and comorbidities of patients are presented in Table 1. A total of 125 (67.6%) female patients hospitalized due to UTI were included in the study. The patients’ age at hospital admission ranged from 21 to 101 years; in total, 133 (71.9%) patients were aged ≥ 65 years (Table 1). Overall, 104 (56.2%) patients had a CCI (Charlson Comorbidity Index) score above 4. The most common comorbidities included cardiovascular diseases (24.9%), diabetes mellitus (24.3%), moderate to severe chronic kidney/liver diseases (13.5% and 13.0%, respectively), and hematological malignant diseases (12.4%) (Table 1). Dementia was also relatively common (9.2%) (Table 1). The majority of patients were discharged home (86.5%), and only a small proportion were admitted to the ICU (3.8%) or another hospital ward (2.2%). The overall 30-day mortality rate was 17 (9.2%) (Table 1), comprising 13 (76.5%) in-hospital deaths and 4 (23.5%) post-discharge deaths.

### 2.2. Diagnosis of UTIs

In most cases, the diagnosis recorded by clinicians differed from that defined according to EAU guideline criteria (Table 2). Among the 185 patients, the rate of misdiagnosis was 24.9% (*n* = 46), and 14.1% (*n* = 26) had ABU (asymptomatic bacteriuria) receiving an unnecessary antibiotic course. In 2.7% (*n* = 5), the UTI diagnosis was not confirmed either by clinical or microbiological tests. As regards non-specific symptoms, in the category of misdiagnosed UTI, confusion occurred in 22.1% (17/77) and was frequently associated with dehydration or dizziness, while dementia occurred in 3.9% (3/77). A total of 84.4% (65/77) of these therapies were initiated at the ED (emergency department), of which 66.2% (43/65) lasted more than three days (6.06 ± 3.96, median 5 days).

Uncategorized UTIs were the most common recorded diagnoses (148/185, 80.0%) when no further classifications of UTI could be found in the patient’s medical records. The highest rate of misdiagnosis (38.9%, 72/185) occurred in uncategorized UTIs (Table 2).

In all misdiagnosed UTI cases, no UTI-specific clinical symptoms were present. The diagnosis of urosepsis recorded at admission (18/185, 9.7%) in five cases (5/18) was not confirmed either by clinical or microbiological tests (Table 2). Therefore, further analyses for empirical antibiotic therapy were only performed for EAU-based ucUTIs and cUTIs, representing 58.4% (108/185) of all patients admitted with UTIs.

### 2.3. EAU-Confirmed UTIs and Most Frequently Isolated Pathogen Species

The most frequently isolated pathogen species by UTI type are presented in Table 3. The incidence of ucUTIs was higher (60/108, 55.6%) than that of cUTIs (48/108, 44.4%). A total of 50.9% (55/108) of patients underwent a urine culture test; out of these, 39.8% (43/108) were positive, and 2.8% (3/108) of the samples were unsuitable for antibiotic sensitivity testing due to suspected contamination. The most common isolated pathogens were *Escherichia coli* (19/43, 44.2%), *Klebsiella* species (6/43, 14.0%), *Enterococcus* species and other *Enterobacteriaceae* species (for both 5/43, 11.6%), and there was no significant difference between UTI types. MDR (multidrug-resistant bacteria) represented only 7.0% (3/43) of all cases. There was more than one pathogen present in the samples of 18.6% (8/43) of the patients (Table 3).

### 2.4. Antibiotic Therapy for EAU-Confirmed UTIs

The characteristics of first antibiotic therapies and key outcomes are described in Table 4. The majority of treatments (75.9%) were monotherapies; in total, 66 (61.1%) patients received their first antibacterial therapy intravenously. The total duration of antibiotic therapy was 6.59 ± 5.21 days. Most patients (69/108, 63.9%) received short-term (1–6 days) antibiotic therapy, while 12.0% (13/108) of patients received prolonged (more than 10 days) antibiotic therapy. However, in the case of ucUTIs, the average duration of antimicrobial therapy was shorter (5.44 ± 3.36, median 4 days) than in the case of cUTIs (8.06 ± 6.68, median 6 days) (*p* = 0.083, *t*-test). In the majority of cases, there was no change in the first empirical therapy (67/108, 62.0%). Changes occurred due to sequential antibiotic therapy (6.5%), de-escalation (5.6%), and escalation (25.9%) (Table 4).

### 2.5. Guideline Adherence in EAU-Confirmed UTIs

Guideline adherence rates to EAU guidelines are presented in Table 4 and Table 5. The initial empirical therapies for UTI showed a relatively low rate (45.4%, 49/108) of guideline adherence in terms of agent selection. Among patients receiving monotherapy (82/108, 75.9%), amoxicillin/clavulanic acid was the most widely used guideline adherent antibiotic agent (43.9%), followed by ciprofloxacin (18.3%) and ceftriaxone (14.6%). There was no guideline for adherent combination therapy. The most common guideline-non-adherent therapies were the combination of these agents with metronidazole (15.4%, 7.7%, and 26.9%, respectively).

Furthermore, overall (agent, route of administration, dose, duration) guideline adherence was found to be substantially lower (10.2%, 11/108) than guideline adherence in terms of agent selection (Table 4). Dosage appropriateness assessment showed a guideline adherence (agent, route of administration, and dose) rate of 36.1% (39/108) while underdosing resulting from higher body weight was 9.3% (10/108) (Table 4).

Regarding the duration of empirical antibiotic treatment, we found a median of 3 days for uc-cystitis, which was in line with the guideline. For uc-pyelonephritis and cUTIs, however, a median of 4 and 6 days, respectively, was found instead of the 7–10 days recommended by the guideline. Overall, the duration of antibiotic therapies was shorter than recommended by the EAU guideline (7–10 days) [11].

## 3. Discussion

Despite the fact that urinary tract infections are among the most prevalent acute infections, this is the first field study in Hungary to evaluate antibiotic prescription patterns, relationships between guideline adherence, and outcomes in patients hospitalized with UTIs. Our main results show that 41.6% of patients failed to meet EAU-based UTI diagnostic criteria and the initial empirical therapies for UTI showed a low rate (45.4%) of guideline adherence regarding agent selection, while the overall guideline adherence was found to be substantially low (10.2%).

### 3.1. Diagnosis

UTIs are the second most common clinical reason for antibiotic treatment in ambulatory and hospital care [9,10,14,20]. The EAU guideline recommends the diagnosis of UTIs should be based on full clinical history (incontinence, diabetes, and other risk factors, etc.) and the presence of clinical symptoms (dysuria, urgency, frequency, chills/fever, flank or suprapubic pain, confusion) [11,21].

Misdiagnoses of UTIs are a growing concern worldwide [22,23]. In UK hospitals, an estimated 40% of cases of UTIs in over 65-year-olds were misdiagnosed, leading to over-prescribing antibiotics, and contributing to the emergence of antibiotic resistance [22,24]. According to the literature, UTIs are commonly misdiagnosed in ED, resulting in unnecessary antibiotic use and delay in the real diagnosis and discharge [14,15,22]. Additionally, empirical antibiotic therapies for misdiagnosed UTIs initiated at the ED are typically continued after admission, which is a further concern [25].

A cohort study conducted at an ED found that on the basis of the combination of symptoms and/or positive urine cultures, only 15% of the recorded UTIs met the UTI diagnosis criteria [16]. Furthermore, in a cohort study conducted in 46 hospitals in Michigan, 27.8% (11.0% to 44.6%) of patients were misdiagnosed with UTI, 81.8% of whom started empirical antibiotic therapy at the ED, and the therapy remained unchanged even after admission [14]. In another multihospital cohort study, in 54.2% of patients misdiagnosed with UTIs, the empirical antibiotic therapy was initiated at the ED, 81.3% of whom had therapy for three or more days [25].

Our results support these findings by showing a total of 38.9% (72/185) misdiagnosed UTIs with unnecessary antibiotic therapy.

Patients are often misdiagnosed with UTI when they are asymptomatic or have symptoms of non-infectious origin, such as lack of appetite or foul-smelling urine [25,26,27,28]. Despite other potential causes, confusion as a non-specific symptom (a common accompanying symptom in urosepsis) is also considered to be the most common reason for suspecting a UTI, which leads to misdiagnosis [29]. Furthermore, false-positive urine tests without specific UTI symptoms might result in UTI misdiagnosis [16,22].

In our study, we found that in all misdiagnosed UTI cases, no UTI-specific clinical symptoms were present. The most common symptom that may have resulted in misdiagnosis was confusion, frequently associated with dehydration or dizziness (17/77, 22.1%) or dementia (3/77, 3.9%). At the same time, in 33.8% (26/77) of the cases, bacteriuria was present, which should have been diagnosed as ABU, but was treated inappropriately with antibiotics, instead.

ABU still remains a challenge to fight since it is commonly treated unnecessarily with antibiotics [30]. In a tertiary care institutional study, ABU could be distinguished from UTI by only 33.7% of resident physicians [31]. In a multihospital cohort study, 31.9% of patients with bacteriuria had ABU, with 78.3% of them receiving unnecessary antibiotic therapy [25]. In a multicenter prospective study, 62% of the patients diagnosed with ABU received an antibiotic therapy previously [32].

Furthermore, in our research, the diagnoses of urosepsis in 5 (5/18) cases could not be confirmed due to missing data, while in the other cases, 13 cases diagnosed on the basis of the EAU guideline were other types of UTIs (Table 2). Studies have shown that urosepsis accounts for 20–30% of all sepsis cases and may worsen rapidly, and inappropriate treatment may even lead to death [33]. This fear may result in overdiagnosis of urosepsis, and inappropriate antibiotic use in hospital settings.

### 3.2. Guideline Adherent Empirical Antibiotic Therapy

Based on the 2015 EAU guideline, empirical antibiotic therapy should be different in uncomplicated and complicated UTIs (Appendix A Table A1) [11]. In the case of the empirical treatment of complicated UTIs with systemic symptoms requiring hospitalization, the recommended initial route of administration is the intravenous one [11]. Moreover, the guideline recommends the use of fluoroquinolones as monotherapy only when local resistance and patient risk factors are considered. The proposed agents are capable of covering *Escherichia coli*, the predominant pathogen in uncomplicated UTIs, and other *Enterobacterales* and *Enterococcus* spp. responsible for complicated UTIs.

#### 3.2.1. Guideline Adherence: Agent Selection

The rate of guideline adherence for antibiotic selection was relatively low—45.4% (49/108). In the study period, national resistance surveillance data from urine samples of hospitalized patients reported the following susceptibility rates in *Escherichia coli:* 71.5% to amoxicillin/clavulanic acid, 70.0% to ciprofloxacin, and 83.3% to ceftriaxone. Additionally, amoxicillin/clavulanic acid and ciprofloxacin showed potent activity (99.2% and 74.2%, respectively) against *Enterococcus faecalis* strains (Table 3), but not for *Enterococcus faecium* (1.6% and 6.1%, respectively. However, *Enterococcus faecium* has not been isolated in this study. *Klebsiella pneumoniae* showed a lower susceptibility for amoxicillin/clavulanic acid (66.1%), ceftriaxone (68.2%), and ciprofloxacin (66.8%) [34].

Inappropriate use of *metronidazole* in combination with other antibacterial was common in our study (a total of 69.2%, 12/26 of combination therapies), despite that metronidazole is ineffective in UTI and not recommended by guidelines. Metronidazole is effective in infections caused by anaerobic bacteria and certain parasites. At the same time, there are hardly any studies reporting UTIs caused by anaerobic bacteria. In the present study, we could not find any rationale for the use of metronidazole in combination therapy. It may be explained with established, but non-evidence-based, malpractice, or, in some cases, the suspicion of concomitant bacterial vaginosis in women.

Considering the route of administration, more than half of the patients (66/108, 66.1%) received intravenous initial antibacterial therapy for UTI. The most common guideline-non-adherent route of administration occurred for the empirical treatment of cUTIs when amoxicillin/clavulanic acid was administered orally instead of intravenously (19/49, 38.78%).

Guideline adherent empirical antibiotic use in UTI varies in the relevant literature. A retrospective cohort study in Jordan evaluating patients hospitalized with ucUTIs found guideline adherent antibiotic therapy in 40% of the cases [35]. In another retrospective observational study conducted at a French ED, the guideline adherence rate was 44% [36]. In addition, a study performed in three general medical wards in New Zealand found only 34% guideline adherence [37]. These rates show that our results are comparable to international findings.

#### 3.2.2. Guideline Adherence: Dosage and Duration of Antibiotic Therapy

The dosage of antibiotic agents is of paramount importance in hospitalized patients. Guideline adherence regarding choice of agent(s), route of administration, and dosing (36.1%) was relatively low. Underdosing affected (9.3%) of UTI patients. The most common underdosing was related to amoxicillin/clavulanic acid when dose adjustment would have been required due to patients’ body weights (Appendix B Table A2). In case of impaired renal function, the administered doses were found to be adequate in all cases.

Although appropriate dosage is important in optimizing antibiotic use and reducing ABR, studies dealing with antibiotic dosing are rare. In a cohort study conducted in a long-term care facility, the risk of *Clostridioides* (previously *Clostridium*) *difficile* infection increased by 94% with suboptimal antibiotic dose [38]. Another retrospective cohort study of uncomplicated UTIs highlighted that the suboptimal doses in ucUTIs increase the prevalence of antibiotic resistance [39].

The average duration of antibiotic therapy for UTIs was 6.59 ± 5.21 days. However, overall (agent, route of administration, dose, duration) guideline adherence was found to be substantially low (10.2%, 11/108). In addition, our results showed that the duration of antibiotic treatment is appropriate in uc-cystitis (median 3 days) and is shorter in uc-pyelonephritis and cUTIs (median 4 and 5.5 days instead of 7–10 days) than recommended (7–10 days).

According to the EAU guideline, the optimal duration of antibiotic therapy varies between different types of UTIs.

In inpatient settings, a small number of studies have addressed the appropriate duration of antibiotic therapy in UTIs. In a study conducted at an American internal medicine clinic, antibiotic selection was appropriate in 97.6% of ucUTIs and 90.5% of cUTIs, but the duration was guideline adherent in 71.96% and 58.6% of cases [40]. This rate is much higher than the one we found in the present study. Moreover, a longer duration of antibiotic therapy increases the risk of *Clostridioides difficile* infection [41,42]. In a meta-analysis of 32 trials for uc-cystitis, a longer duration of antibiotic therapy was associated with more adverse effects, but with a lower risk of clinical outcome failure [41]. A Cochrane database systematic review found no difference in efficacy between shorter (3–6 days) and longer (7–14 days) therapies in ucUTIs in elderly people [43]. Furthermore, the EAU guideline recommends a longer duration of antibiotic therapy for male patients. Nevertheless, a recent randomized controlled trial found that the 7-day therapy was not inferior compared to the 14-day therapy [44]. In addition, prolonged UTI treatment may contribute to the emergence of ABR [45].

Based on these facts, the optimal duration of antibiotic therapy in UTIs is not well defined.

### 3.3. Changes in the First Empirical Therapy

Based on the EAU guideline, switching from an intravenous to an oral regimen should be based on the improvement of clinical symptoms when either the same agent or the same drug class should be used [11].

Our results show that switching from an IV to an oral regimen (in 6.5% of the cases) was performed within a median of 2 (1–12) days.

Based on the results of two retrospective cohort studies, iv switching to po treatment was associated with a shorter LOS, shorter antibiotic duration, lower antibiotic exposure, and direct antibiotic costs, compared to iv therapy. Moreover, there was no difference in clinical outcomes (failure, readmission rate, mortality rate) between the two groups [46,47]. To sum up, iv switching to po within an appropriate time may improve the rational use of antibiotics and clinical outcomes.

In this study, antibiotic therapies were escalated quite often (28/108, 25.9%), and in 18 cases due to the antibiogram. De-escalation (6/108, 5.6%) occurred at relatively low rates. According to a retrospective cohort single-center study, de-escalation was associated with better patient outcomes (e.g., reduced LOS), and MDR pathogens were the only significant reason identified for de-escalation failure [48]. However, we found no significant difference in LOS in patients with or without de-escalation. Antibiotic de-escalation after the first 48 h based on susceptibility testing could be an essential antimicrobial stewardship strategy without an increase in hospital length of stay or patients’ mortality [49].

### 3.4. Strengths and Limitations

This primary data collection enabled an in-depth analysis of antibiotic use in the empirical treatment of UTIs at the Internal Medicine Department of a University Hospital. Nevertheless, retrospective data collection from medical records might contain inaccuracies, missing values, and potential biases.

Another limitation of this study was that there were no available written protocols at the national and hospital level (considering local resistance patterns), regarding the diagnosis and empirical antibiotic therapy of UTIs. Moreover, contrary to international practice, fluoroquinolones were considered guideline adherent agents. However, according to the national surveillance data published later, the sensitivity of the most common UTI pathogen (*Escherichia coli*) to these antibiotics was low (70.0%) during the data collection period. Therefore, the guideline adherence rate is overestimated. Also, there was a lack of data regarding the antibiotic prescriptions at discharge from the hospital, which were not recorded in this study.

Finally, this single-center study evaluated the prescribing patterns in one university tertiary care center, and results may not be extrapolated to other ones.

## 4. Materials and Methods

### 4.1. Study Design and Setting

This 1-year (January–December 2017) retrospective observational study is part of a larger retrospective observational study conducted in a 110-bed internal medicine unit of the University of Debrecen [50,51].

### 4.2. Data Collection

Data collection was the same as the one described in our previous study [51]. The ward pharmacist collected data for all patients who received antibiotic therapy during hospital stay. Using the Hospital Information System (e-MedSolution, later UDMED, Debrecen, Hungary), all patient and therapy-related data were manually collected from medication charts and discharge letters, such as age, sex, weight, date of hospital admission and discharge, comorbidities, and type of discharge. On the day of admission, laboratory test results (white blood cell count, CRP, eGFR, or estimated glomerular filtration rate), urine culture tests and microorganisms isolated from samples were also recorded, along with clinical outcome (30-day mortality). Prehospital antibiotic therapy, medication allergies, indications for antibiotic treatment, empirical antibiotic choice, dose, mode of administration, and length of antibacterial therapy during hospital stay were among the data collected for antibacterial therapy. Spreadsheets for Microsoft Excel were used to insert the extracted data for additional analysis.

The study included only adult (18 years or older) patients who received empirical antibiotic therapy for urinary tract infection. In this investigation, the classification of UTI (risk factors, signs and symptoms, and laboratory diagnosis) was performed according to the 2015 EAU guideline, which is shown in Table A1.

This guideline uses the concept of uncomplicated and complicated UTIs, targets adult patients (over 18 years), includes the algorithm for the diagnosis of UTIs, and provides recommendations for the oral and intravenous empirical antibiotic treatment of ucUTIs (uncomplicated UTIs), cUTIs (complicated UTIs), CA-UTIs (catheter-associated UTIs), and US (urosepsis) [11].

Antibacterial therapy without pathogen identification and susceptibility testing was referred to as empirical treatment. Charlson Comorbidity Index (CCI) was used to assess the general health status of the patients [52]. The appropriateness of the drug dosage for antibacterials excreted renally was evaluated using eGFR upon admission. The ATC/DDD index (version 2023) of the World Health Organization was used to determine the extent of antibiotic exposure. The assumed average maintenance dose per day in adults is referred to as the defined daily dose (DDD). DDD, in terms of antibiotics, refers to infections with moderate severity [53]. We analyzed systemic antibacterials (ATC code: J01). The term LOS (length of stay) describes how long a patient stays in the hospital. The days of admission and discharge were counted separately as one day.

### 4.3. Main Outcome Measures

The primary outcome measure was to assess whether recorded diagnoses by clinicians met the EAU 2015 guideline-based diagnosis criteria [11]. Therefore, diagnosis was assessed based on clinical presentation, microbiological results, and the grade of severity of the infection, also considering risk factors. Diagnosis was considered misdiagnosis when not confirmed by clinical presentation or clinical signs and symptoms. Adherence to the EAU guideline with regard to the selection of empirical antibiotic(s) and dosage was one of the secondary outcome variables.

Assessment for guideline adherence was performed as follows:

*Choice assessment:* The initial empirical antibiotic treatment started for hospitalized patients with UTI was classified as adherent or non-adherent based on antibiotic selection. When all the antibacterial agents in the combination adhered to the guidelines, the combined therapy was deemed adherent.

*Dosage assessment:* Based on the previously described guideline, we defined the dose of the first adherent empiric antibiotic therapy as follows:

Appropriate dose: the amount advised by the guidelines, plus dose modification in cases of renal impairment.

Under or overdose: the amount taken in excess of the guidelines and/or no dose modification in cases of renal impairment and in extremes for body weight.

Summary of product characteristics (SPC), which provides a complete description of how to take body weight and eGFR into account in dose calculation, was also taken into account in cases of extreme body weight (<40 and >100 kg) and impaired renal function. When it came to the choice of antibiotic, therapies deemed non-adherent did not undergo dosage assessment.

Furthermore, modifications to the initial antimicrobial treatment were evaluated (sequential therapy: moving from an iv to an oral regimen, de-escalation, or escalation). De-escalation was defined as reducing the spectrum; escalation of the antibiotic regimen was described as introducing a new antibiotic or switching to a broad-spectrum antibacterial.

### 4.4. Statistical Analyses

Descriptive statistics such as frequency, mean and standard deviation were computed to describe the variables of the study. Differences in continuous variables such as duration of antimicrobial therapy were assessed with a two-sample *t*-test (and nonparametric *t*-test). Statistical analyses were performed using SPSS software version 29 (SPSS Inc., Chicago, IL, USA), values of *p* < 0.05 were considered statistically significant.

Patients were anonymized and thus made unidentifiable in the study.

## 5. Conclusions

We found a relatively high rate of misdiagnosed UTIs in this study, which provides further evidence for the fact that antimicrobial stewardship interventions for UTIs in hospital wards are crucial. These results also draw attention to the need for improvement of empirical prescribing by implementing of antibiotic stewardship program, and through this, limiting unnecessary combinations and optimizing the dosage. We believe that our findings may help to optimize diagnosis and antibiotic use in UTIs.

## Figures and Tables

**Table 1:** Demographic and clinical characteristics of patients with UTI.

Parameters	*N*	%
	185	100
Gender (female)	125	67.6
Age (years) (mean ± SD, range)	71.87 ± 15.04 (21–101)	
20–64 years	52	28.1
65+ years	133	71.9
CCI (Charlson Comorbidity Index) (mean ± SD, range)	4.71 ± 1.96 (0–11)	
0	7	3.78
1	1	0.5
2	12	6.5
3	28	15.1
4	33	17.8
>4	104	56.2
Comorbidities		
Cardiovascular disease *	46	24.9
Diabetes mellitus	45	24.3
Chronic kidney disease (moderate to severe)	25	13.5
Chronic liver disease (moderate to severe)	24	13.0
Hematological malignant diseases	23	12.4
Solid tumor	18	9.7
Localized	16	8.6
Metastatic	2	1.1
Dementia	17	9.2
Cerebrovascular accident or transient ischemic attack	15	8.1
Peptic ulcer disease	14	7.6
Chronic obstructive pulmonary disease	8	4.3
Peripheral vascular disease	1	0.5
Discharge types		
Discharged home	160	86.5
Other hospital ward	4	2.2
ICU (intensive care unit)	7	3.8
LTCF (long-term care facility)	1	0.5
Outcome		
In-hospital mortality	13	7.0
30-day mortality	17	9.2

SD: standard deviation; *: congestive heart failure, ischaemic heart diseases, or arrhythmias.

**Table 2:** Comparison of diagnosis recorded at hospital admission and EAU diagnosis.

Recorded Diagnosis	EAU-Confirmed Diagnosis
**Total (*n* = 185), 100%**	
Cystitis (*n* = 10), 5.4%	ucUTI (*n* = 3) cUTI (*n* = 5) ABU (*n* = 1) Misdiagnosis (*n* = 1)
Pyelonephritis (*n* = 9), 3.8%	ucUTI (*n* = 4) cUTI (*n* = 3) Misdiagnosis (*n* = 2)
Urosepsis (*n* = 18), 9.7%	ucUTI (*n* = 7) cUTI (*n* = 4) ABU (*n* = 2) Unconfirmed urosespsis (*n* = 5)
UTI (*n* = 148), 80.0%	ucUTI (*n* = 46) * cUTI (*n* = 36) ABU (*n* = 23) Misdiagnosis (*n* = 43)
	Total: ucUTI (*n* = 60) *, 32.4% cUTI (*n* = 48), 25.9% ABU (*n* =26), 14.1% Unconfirmed urosepsis (*n* = 5), 2.7% Misdiagnosis (*n* = 46), 24.9%

EAU: European Association of Urology; ucUTI: uncomplicated urinary tract infection with pyelonephritis; cUTI: complicated urinary tract infection with pyelonephritis; ABU: asymptomatic bacteriuria; UTI: urinary tract infection; * one uncomplicated cystitis.

**Table 3:** Most frequently isolated pathogen species in EAU-confirmed UTIs found in this study.

Pathogens	ucUTI (Cystitis) *N* = 1	ucUTI (Pyelonephritis) *N* = 59 (%)	cUTI (Pyelonephritis) *N* = 48 (%)	Total *N* = 108 (%)
Total urine cultures	-	30 (50.8%)	25 (52.1%)	55 (50.9%)
Positive urine cultures	-	23 (39.0%)	20 (41.7%)	43 (39.8%)
Contaminated sample	-	2 (3.4%)	1 (2.1%)	3 (2.8%)
From positive urine cultures (%)				
More than one pathogen per sample	-	6 (26.12%)	2 (10.0%)	8 (18.6%)
*Esherchia coli*	-	10 (43.5%)	9 (45.0%)	19 (44.2%)
*Klebsiella* spp.	-	4 (17.4%)	2 (10.0%)	6 (14.0%)
Other *Enterobacteriaceae* spp.	-	4 (17.4%)	1 (5.0%)	5 (11.6%)
*Enterococcus faecalis*	-	1 (4.3%)	4 (20.0%)	5 (11.6%)
*Pseudomonas* spp.	-	1 (4.3%)	3 (15.0%)	4 (9.3%)
*Proteus* spp.	-	1 (4.3%)	-	1 (2.3%)
*Streptococcus* spp.	-	1 (4.3%)	-	1 (2.3%)
*Candida* spp.	-	4 (17.4%)	3 (15.0%)	7 (16.3%)
MDR *bacteria*	-	3 (13.0%)	-	3 (7.0%)

ucUTI: uncomplicated urinary tract infection; cUTI: complicated urinary tract infection; MDR: multidrug-resistant (MDR) (bacteria with acquired resistance to at least three antibiotic subgroups).

**Table 4:** Characteristics of antibiotic therapies for EAU-confirmed UTIs.

Parameters	*N*	%
	108	100
EAU guideline-adherent agent(s)	49	45.4
EAU guideline adherent agent(s) and route of administration	49	45.4
EAU guideline adherent agent(s), route of administration, and dose	39	36.1
EAU guideline adherent agent(s), route of administration, dose, and duration	11	10.2
Type of the first antibiotic therapy		
Monotherapies	82	75.9
Combination therapies	26	24.1
Route of administration of the first antibiotic therapy		
intravenous	66	61.1
oral	42	38.9
Dosage of the first antibiotic therapy for guideline adherent agent selection		
appropriate	39	36.1
overdosed (compared to SPC due to lack of guideline recommended dose)	0	0.0
underdosed (compared to EAU guideline and due to body weight)	10	9.3
Duration of total antibiotic therapies (Mean ± SD, range) 6.59 ± 5.21 (1–35)		
Total antibiotic exposure (Mean ± SD, DDD/patient) 9.84 ± 14.99 (0.50–72.47)		
Number of consecutive antibiotic therapies		
1	69	63.9
>1 (2–5)	39	36.1
Changes in the first empirical therapy		
Sequential antibiotic therapy *	7	6.5
De-escalation	6	5.6
Escalation	28	25.9
No change	67	62.0

EAU: European Association of Urology; iv: intravenously; SPC: summary of product characteristics; SD: standard deviation; * switch from an iv to oral regimen.

**Table 5:** The distribution of first empirical antibiotic therapies (mono- and combination therapies) in EAU-confirmed UTIs.

Antibiotics	Route of Administration ^4^	Doses/Day	Frequency (N)	Total %		EAU Guideline Adherence ^4^				
					Guideline Adherence %	uc-Cystitis (*n* = 1)	uc-Pyelonephritis (*n* = 59)	cUTI without Pyelonephritis (*n* = 0)	cUTI with Pyelonephritis (*n* = 48)	Rosepsis (*n* = 0)
		Monotherapies (*N* = 82; 100%)								
Fosfomycin trometamol	po	3 g q.d	1	1.2	0.0		1			
Nitrofurantoin monohydrate	po	100 mg b.i.d-t.i.d	2	2.4	0.0		1		1	
Amoxicillin/clavulanic acid	po	625 mg b.i.d-t.i.d	12	14.6	11.0	1	9		2	
Amoxicillin/clavulanic acid	iv	1.2 g b.i.d-t.i.d	24	29.3	29.3		14		10	
Ceftibuten	po	-	0	0.0	0.0					
Cefuroxim axetil	po	-	0	0.0	0.0					
Cefuroxim sodium	iv	-	0	0.0	0.0					
Trimethoprim-sulphamethoxazole ^1^	po	400–800/80–160 mg b.i.d	3	3.7	0.0				3	
Ofloxacin	po	-	0	0.0	0.0					
Norfloxacin	po	400 mg b.i.d	3	3.7	1.2		1		2	
Ciprofloxacin ^2^	po	500 mg b.i.d	12	14.6	14.6		7		5	
Ciprofloxacin ^2^	iv	400 mg b.i.d	3	3.7	3.7		1		2	
Levofloxacin ^2^	po	500 mg q.d	2	2.4	2.4		2			
Levofloxacin ^2^	iv	-	0	0.0	0.0					
Cefotaxime	iv	-	0	0.0	0.0					
Ceftriaxone	iv	2 g q.d	12	14.6	14.6		5		7	
Cefepime	iv	-	0	0.0	0.0					
Piperacillin/tazobactam	iv	-	0	0.0	0.0					
Imipenem/cilastatin	iv	-	0	0.0	0.0					
Meropenem	iv	500 mg t.i.d- 1 g b.i.d	4	4.9	2.4		2		2	
Clarithromycin	po	500 mg b.i.d	1	1.2	0.0				1	
Gentamicin monotherapy	iv	80–120 mg q.d	2	2.4	0.0		2			
Moxifloxacin	po	400 mg q.d	1	1.2	0.0				1	
		Combination therapies (*N* = 26; 100%)								
Amoxicillin-clavulanic acid + amikacin/gentamicin ^3^	iv	1.2 g t.i.d + 80 mg b.i.d	1	3.8	0.0		1			
Ceftriaxone + amikacin/gentamicin ^3^	iv	-	0	0.0	0.0					
Piperacillin/tazobactam + amikacin/gentamicin ^3^	iv	-	0	0.0	0.0					
Meropenem + amikacin/gentamicin ^3^	iv	-	0	0.0	0.0					
Imipenem-cilastatin + amikacin/gentamicin ^3^	iv	-	0	0.0	0.0					
Amoxicillin-clavulanic acid + clarithromycin	iv	1.2 g t.i.d + 500 mg b.i.d	1	3.8	0.0				1	
Amoxicillin-clavulanic acid + metronidazole	iv + po	1.2 g b.i.d-t.i.d + 500 mg b.i.d	4	15.4	0.0		1		3	
Amoxicillin-clavulanic acid + metronidazole	po	625 mg b.i.d-t.i.d + 500 mg b.i.d	2	7.7	0.0		2			
Amoxicillin-clavulanic acid + Trimethoprim-sulphamethoxazole	iv + po	1.2 g t.i.d + 400/80 mg b.i.d	1	3.8	0.0		1			
Ceftriaxone + metronidazole	iv	2 g q.d + 250–500 mg b.i.d	5	19.2	0.0		4		1	
Ceftriaxone + metronidazole	iv + po	2 g q.d + 500 mg b.i.d	2	7.7	0.0				2	
Ceftriaxone + trimethoprim-sulphamethoxazole	iv + po	500 mg b.i.d–2 g q.d + 400/80 mg b.i.d	2	7.7	0.0		1		1	
Ciprofloxacin + metronidazole	po	500 mg b.i.d + 500 mg b.i.d	2	7.7	0.0		1		1	
Ciprofloxacin + moxifloxacin	iv + po	500 mg b.i.d + 400 mg q.d	1	3.8	0.0		1			
Ciprofloxacin + tobramycin	iv	500 mg b.i.d + 80 mg b.i.d	1	3.8	0.0				1	
Nitrofurantoin + metronidazole	po	100 mg t.i.d + 500 mg b.i.d	2	7.7	0.0		1		1	
Imipenem + metronidazole	iv + po	1 g t.i.d + 500 mg b.i.d	1	3.8	0.0				1	
Tygecyclin + colomycin	iv	400 mg q.d + 1 IU t.i.d	1	3.8	0.0		1			

Green color: First-line guideline-adherent treatment, Orange color: Second-line guideline treatment (in case of failure of initial therapy within 1–3 days), No Color: Guideline-non-adherent treatment. UTIs: urinary tract infections; EAU: European Association of Urology; uc: uncomplicated; cUTI: complicated urinary tract infection; iv: intravenous; po: per oral. ^1^ When trimethoprim-sulphamethoxazole resistance is less than 20%. ^2^ Considering fluoroquinolone local resistance and patients risk factors. ^3^ In combination with other antimicrobials (e.g., amino- and acylaminopenicillin with beta lactamase inhibitor, cephalosporins, carbapenems). ^4^ Patients with systemic symptoms requiring hospitalization should be initially treated with an intravenous antimicrobial regimen; b.i.d: twice daily; t.i.d: three times daily; q.d: every day.

## Data Availability

Data are available from the corresponding author upon reasonable request. The data are not publicly available due to due to privacy and ethical restrictions.

## Appendix A

**Table A1:** Classification of UTIs in this study, risk factors, possible signs and symptoms, and diagnosis.

Types of UTIs	uc-Cystitis	uc-Pyelonephritis	cUTI without Pyelonephriti	cUTI with Pyelonephritis	Urosepsis
Risk Factors					
No known relevant anatomical and functional abnormalities within the urinary tract, indwelling urinary catheters, renal diseases, obstruction	✓	✓			
No comorbidities ^1^	✓	✓			
Age (over 65 years old)			✓	✓	
Pregnancy			✓	✓	
Men			✓	✓	
Known relevant anatomical and functional abnormalities within the urinary tract, indwelling urinary catheters, renal diseases, obstruction			✓	✓	✓
Comorbidities ^1^			✓	✓	✓
Recent history of HAI ^2^			✓	✓	✓
SIRS, life-threatening organ dysfunction ^3^					✓
Potential signs and symptoms					
Lower urinary tract: dysuria, frequency, urgency	✓	✓	✓	✓	
Absence of vaginal discharge or irritation	✓				
Fever (>38 °C)		✓	✓	✓	✓
Hypothermia					✓
Chills, suprapubic pain		✓	✓	✓	
Chills, flank pain				✓	
Nausea, vomiting		✓	✓	✓	
Costovertebral angle tenderness		✓	✓	✓	
Bacteriuria, pyuria			✓	✓	✓
Tachycardia and tachypnoea					✓
Differential diagnosis					
Imaging technique ^4^		✓	✓		
Laboratory diagnosis					
Urinalysis (assessment of white and red blood cells and nitrite) ^5^		✓	✓		
Urine culture and antimicrobial susceptibility testing ^5^		✓	✓	✓	✓
Blood culture					✓
CBC (leukocytosis or leukopenia)					✓
Rise in the level of PCT					✓
Rise in the level of Serum lactate					✓

✓: the presence of the indicator in a certain UTI group. uc: uncomplicated; cUTI: complicated urinary tract infection; SIRS: systemic inflammatory response syndrome; CBC: complete blood count; PCT: procalcitonin. ^1^ concomitant immunocompromising diseases, e.g., diabetes. ^2^ HAI: healthcare-associated infections (e.g., isolated ESBL-producing organisms, isolated multi-drug resistant organisms). ^3^ Caused by a dysregulated host response to infection originating from the urinary tract and/or male genital organs. ^4^ Ultrasound (US): to rule out urinary tract obstruction or renal stone disease in patients with a history of urolithiasis, renal function disturbances or a high urine pH; contrast-enhanced computed tomography (CT) scan, or excretory urography: if the patient remains febrile after 72 h of treatment, or immediately if there is deterioration in clinical status; magnetic resonance imaging (MRI): to avoid radiation risk to the fetus. ^5^ In the case of uncomplicated cystitis, it only leads to a minimal increase in diagnostic accuracy; however, it is recommended in patients with atypical symptoms, as well as those who fail to respond to appropriate antimicrobial therapy. Renal insufficiency: dose adjustment is needed when glomerular filtration rate (GFR) is <20 mL/min, with the exception of antimicrobials with nephrotoxic potential (e.g., aminoglycosides).

## Appendix B

**Table A2:** Dosage assessment parameters.

Antimicrobial Agent	Appropriate (Recommended) Dose	Recommended Dose Adjustment by SPC		Debatable Dose	Underdose/Overdose
	EAU UTI Guideline ^1^	eGFR (mL/min)	Body Weight (kg)		
Fosfomycin trometamol	oral 3 g SD	<10		<50% deviation from the recommended dose and/or absence of loading dose	≥50% deviation from the recommended dose and/or no dose adjustment in renal impairment and in extremes ^2^ for body weight
Nitrofurantoin monohydrate	oral 100 mg b.i.d	≤50			
Amoxicillin/clavulanic acid	oral 375 mg t.i.d	<10	<50 kg		
Amoxicillin/clavulanic acid	oral 625 mg t.i.d	<10	<50 kg		
Amoxicillin/clavulanic acid	iv 1.2 g t.i.d	<10	<50 kg		
Cefuroxim axetil	oral 500 mg b.i.d	<20			
Cefuroxim sodium	iv 0.75–1.5 g b.i.d-t.i.d	<20			
Trimethoprim/sulphametoxazole	oral 160/800 mg b.i.d	<30			
Ofloxacin	oral 200 mg b.i.d	<50			
Norfloxacin	oral 400 mg b.i.d	<20			
Ciprofloxacin	oral 500–750 mg b.i.d or iv 400 mg b.i.d	<30			
Levofloxacin	oral 750 mg q.d iv 500 mg q.d	<50			
Cefotaxime	iv 2 g t.i.d	<5			
Ceftriaxone	iv 1–2 g q.d	<10	<50 kg		
Cefepime	iv 1–2 g q.d	<50	<40 kg		
Piperacillin/tazobactam	iv 2.5–4.5 g t.i.d	<50			
Gentamicin	iv 5 mg/kg q.d	<40	5 mg/bodyweight		
Amikacin	iv 15 mg/kg q.d	<70	5 mg/bodyweight		
Imipenem/cilastatin	iv 0.5 g t.i.d	<70			
Meropenem	iv 1 g t.i.d	<50			

EAU: European Association of Urology; UTI: urinary tract infection; SPC: summary of product characteristics; eGFR: estimated glomerular filtration rate (MDRD GFR equation); SD: single dose; b.i.d: twice daily; t.i.d: three times daily; q.d: every day; iv: intravenous(ly). ^1^ First empirical antibiotic is recommended to be given intravenously when the patient has cUTI requiring hospitalization. ^2^ Extreme body weights: low body weight defined by SPC, and extreme overweight ≥ 100 kg.

## References

[1] Ghosh D., Veeraraghavan B., Elangovan R., Vivekanandan P. (2020). Antibiotic Resistance and Epigenetics: More to It than Meets the Eye. Antimicrob. Agents Chemother..

[2] Allel K., Day L., Hamilton A., Lin L., Furuya-Kanamori L., Moore C.E., Van Boeckel T., Laxminarayan R., Yakob L. (2023). Global antimicrobial-resistance drivers: An ecological country-level study at the human-animal interface. Lancet Planet. Health.

[3] Antimicrobial Resistance C. (2022). Global burden of bacterial antimicrobial resistance in 2019: A systematic analysis. Lancet.

[4] Mello M.S., Oliveira A.C. (2021). Overview of the actions to combat bacterial resistance in large hospitals. Rev. Lat. Am. Enferm..

[5] Brusselaers N., Vogelaers D., Blot S. (2011). The rising problem of antimicrobial resistance in the intensive care unit. Ann. Intensive Care.

[6] Cortes-Penfield N.W., Trautner B.W., Jump R.L.P. (2017). Urinary Tract Infection and Asymptomatic Bacteriuria in Older Adults. Infect. Dis. Clin. N. Am..

[7] Yang X., Chen H., Zheng Y., Qu S., Wang H., Yi F. (2022). Disease burden and long-term trends of urinary tract infections: A worldwide report. Front. Public Health.

[8] ECDC Antimicrobial Consumption Database (ESAC-Net). https://www.ecdc.europa.eu/en/antimicrobial-consumption/surveillance-and-disease-data/database.

[9] ECDC European Centre for Disease Prevention and Control, Antimicrobial Use in European Hospitals. https://www.ecdc.europa.eu/en/publications-data/antimicrobial-use-european-hospitals.

[10] ECDC Diagnosis Site of Antimicrobial Treatment. https://www.ecdc.europa.eu/en/healthcare-associated-infections-acute-care-hospitals/database/indications-antimicrobial-use/diagnosis-site.

[11] Grabe M., Bjerklund-Johansen T.E., Botto H., Çek M., Naber K.G., Tenke P., Wagenlehner F. Guidelines on Urological Infections. https://d56bochluxqnz.cloudfront.net/documents/EAU-Guidelines-Urological-Infections-2015.pdf.

[12] Ostrow O., Prodanuk M., Foong Y., Singh V., Morrissey L., Harvey G., Campigotto A., Science M. (2022). Decreasing Misdiagnoses of Urinary Tract Infections in a Pediatric Emergency Department. Pediatrics.

[13] Scott V.C.S., Thum L.W., Sadun T., Markowitz M., Maliski S.L., Ackerman A.L., Anger J.T., Kim J.H. (2021). Fear and Frustration among Women with Recurrent Urinary Tract Infections: Findings from Patient Focus Groups. J. Urol..

[14] Gupta A., Petty L., Gandhi T., Flanders S., Hsaiky L., Basu T., Zhang Q., Horowitz J., Masood Z., Chopra V. (2022). Overdiagnosis of urinary tract infection linked to overdiagnosis of pneumonia: A multihospital cohort study. BMJ Qual. Saf..

[15] Tomas M.E., Getman D., Donskey C.J., Hecker M.T. (2015). Overdiagnosis of Urinary Tract Infection and Underdiagnosis of Sexually Transmitted Infection in Adult Women Presenting to an Emergency Department. J. Clin. Microbiol..

[16] Childers R., Liotta B., Brennan J., Wang P., Kattoula J., Tran T., Montilla-Guedez H., Castillo E.M., Vilke G. (2022). Urine testing is associated with inappropriate antibiotic use and increased length of stay in emergency department patients. Heliyon.

[17] Vaughn V.M., Flanders S.A., Snyder A., Conlon A., Rogers M.A.M., Malani A.N., McLaughlin E., Bloemers S., Srinivasan A., Nagel J. (2019). Excess Antibiotic Treatment Duration and Adverse Events in Patients Hospitalized with Pneumonia: A Multihospital Cohort Study. Ann. Intern. Med..

[18] Benko R., Matuz M., Juhasz Z., Bognar J., Bordas R., Soos G., Hajdu E., Peto Z. (2019). Treatment of Cystitis by Hungarian General Practitioners: A Prospective Observational Study. Front. Pharmacol..

[19] Juhasz Z., Benko R., Matuz M., Viola R., Soos G., Hajdu E. (2013). Treatment of acute cystitis in Hungary: Comparison with national guidelines and with disease-specific quality indicators. Scand. J. Infect. Dis..

[20] ECDC Indications for Antimicrobial Use. https://www.ecdc.europa.eu/en/healthcare-associated-infections-acute-care-hospitals/database/indications-antimicrobial-use.

[21] Bonkat G.R.B., Bruyère F., Cai T. EAU Guideline on Urological Infections. https://uroweb.org/guidelines/urological-infections.

[22] Rousham E., Cooper M., Petherick E., Saukko P., Oppenheim B. (2019). Overprescribing antibiotics for asymptomatic bacteriuria in older adults: A case series review of admissions in two UK hospitals. Antimicrob. Resist. Infect. Control.

[23] Armstrong N. (2018). Overdiagnosis and overtreatment as a quality problem: Insights from healthcare improvement research. BMJ Qual. Saf..

[24] Woodford H.J., George J. (2009). Diagnosis and management of urinary tract infection in hospitalized older people. J. Am. Geriatr. Soc..

[25] Vaughn V.M., Gupta A., Petty L.A., Gandhi T.N., Flanders S.A., Swaminathan L., Hsaiky L., Ratz D., Horowitz J. (2020). Misdiagnosis of Urinary Tract Infection Linked to Misdiagnosis of Pneumonia: A Multihospital Cohort Study. Infect. Control Hosp. Epidemiol..

[26] Walker S., McGeer A., Simor A.E., Armstrong-Evans M., Loeb M. (2000). Why are antibiotics prescribed for asymptomatic bacteriuria in institutionalized elderly people? A qualitative study of physicians’ and nurses’ perceptions. CMAJ.

[27] Kistler C.E., Sloane P.D., Platts-Mills T.F., Beeber A.S., Khandelwal C., Weber D.J., Mitchell C.M., Reed D., Chisholm L., Zimmerman S. (2013). Challenges of antibiotic prescribing for assisted living residents: Perspectives of providers, staff, residents, and family members. J. Am. Geriatr. Soc..

[28] Kistler C.E., Wretman C.J., Zimmerman S., Enyioha C., Ward K., Farel C.E., Sloane P.D., Boynton M.H., Beeber A.S., Preisser J.S. (2022). Overdiagnosis of urinary tract infections by nursing home clinicians versus a clinical guideline. J. Am. Geriatr. Soc..

[29] Mayne S., Bowden A., Sundvall P.D., Gunnarsson R. (2019). The scientific evidence for a potential link between confusion and urinary tract infection in the elderly is still confusing—A systematic literature review. BMC Geriatr..

[30] Phillips C.D., Adepoju O., Stone N., Moudouni D.K., Nwaiwu O., Zhao H., Frentzel E., Mehr D., Garfinkel S. (2012). Asymptomatic bacteriuria, antibiotic use, and suspected urinary tract infections in four nursing homes. BMC Geriatr..

[31] Lee M.J., Kim M., Kim N.H., Kim C.J., Song K.H., Choe P.G., Park W.B., Bang J.H., Kim E.S., Park S.W. (2015). Why is asymptomatic bacteriuria overtreated?: A tertiary care institutional survey of resident physicians. BMC Infect. Dis..

[32] Illiano E.B.R., Li Marzi V., Mancini V., Finazzi Agrò E., De Rienzo G., Natale F., Pastore A., Palleschi G., Balzarro M., Rubilotta E. (2018). Urinary tract infection still a challenge to fight: A real setting study. Neurourol. Urodyn..

[33] Zhang L., Zhang F., Xu F., Wang Z., Ren Y., Han D., Lyu J., Yin H. (2021). Construction and Evaluation of a Sepsis Risk Prediction Model for Urinary Tract Infection. Front. Med..

[34] NCE Results of National Microbiological Surveillance Results on Antibiotic Resistance. http://www.oek.hu/oek.web?nid=666&pid=12.

[35] Alrosan S., Al Mse’adeen M., Alkhawaldeh I.M., Mishael J., Aljarab’ah N., Aljarajreh M., Yamin M., Abu-Jeyyab M. (2023). An Audit to Reevaluate the Adherence to the Guidelines in Patients with Urinary Tract Infection at the Al-Karak Hospital in Jordan. Cureus.

[36] Ramdani A., Rebaudet S., Beni-Chougrane N., Penaranda G., Coquet E. (2017). A review of urinary tract infection management for patients admitted to the emergency department: Assessment of adherence to guidelines and identification of hospitalization criteria. Pharm. Hosp. Clin..

[37] Chen A.K., Duffy E.J., Ritchie S.R., Thomas M.G. (2019). Diagnostic accuracy and adherence to treatment guidelines in adult inpatients with urinary tract infections in a tertiary hospital. J. Pharm. Pract. Res..

[38] Appaneal H.J., Shireman T.I., Lopes V.V., Mor V., Dosa D.M., LaPlante K.L., Caffrey A.R. (2021). Poor clinical outcomes associated with suboptimal antibiotic treatment among older long-term care facility residents with urinary tract infection: A retrospective cohort study. BMC Geriatr..

[39] Shafrin J., Marijam A., Joshi A.V., Mitrani-Gold F.S., Everson K., Tuly R., Rosenquist P., Gillam M., Ruiz M.E. (2022). Impact of suboptimal or inappropriate treatment on healthcare resource use and cost among patients with uncomplicated urinary tract infection: An analysis of integrated delivery network electronic health records. Antimicrob. Resist. Infect. Control.

[40] Sigler M., Leal J.E., Bliven K., Cogdill B., Thompson A. (2015). Assessment of Appropriate Antibiotic Prescribing for Urinary Tract Infections in an Internal Medicine Clinic. South. Med. J..

[41] Katchman E.A., Milo G., Paul M., Christiaens T., Baerheim A., Leibovici L. (2005). Three-day vs longer duration of antibiotic treatment for cystitis in women: Systematic review and meta-analysis. Am. J. Med..

[42] Vogel T., Verreault R., Gourdeau M., Morin M., Grenier-Gosselin L., Rochette L. (2004). Optimal duration of antibiotic therapy for uncomplicated urinary tract infection in older women: A double-blind randomized controlled trial. CMAJ.

[43] Lutters M., Vogt-Ferrier N.B. (2008). Antibiotic duration for treating uncomplicated, symptomatic lower urinary tract infections in elderly women. Cochrane Database Syst. Rev..

[44] Drekonja D.M., Trautner B., Amundson C., Kuskowski M., Johnson J.R. (2021). Effect of 7 vs 14 Days of Antibiotic Therapy on Resolution of Symptoms among Afebrile Men with Urinary Tract Infection: A Randomized Clinical Trial. JAMA.

[45] Goebel M.C., Trautner B.W., Grigoryan L. (2021). The Five Ds of Outpatient Antibiotic Stewardship for Urinary Tract Infections. Clin. Microbiol. Rev..

[46] Rieger K.L., Bosso J.A., MacVane S.H., Temple Z., Wahlquist A., Bohm N. (2017). Intravenous-only or Intravenous Transitioned to Oral Antimicrobials for Enterobacteriaceae-Associated Bacteremic Urinary Tract Infection. Pharmacotherapy.

[47] Gamble K.C., Rose D.T., Sapozhnikov J. (2021). Intravenous to Oral Antibiotics Versus Intravenous Antibiotics: A Step-Up or a Step-Down for Extended Spectrum Beta-Lactamase Producing Urinary Tract Infections?. Open Forum Infect. Dis..

[48] Alshareef H., Alfahad W., Albaadani A., Alyazid H., Talib R.B. (2020). Impact of antibiotic de-escalation on hospitalized patients with urinary tract infections: A retrospective cohort single center study. J. Infect. Public Health.

[49] Iffat Shafiq J.M., Schweighardt A., Zak M., Evans C. (2016). Incidence of Antibiotic De-Escalation in Hospitalized Internal Medicine Patients Diagnosed with Cystitis or Pyelonephritis. Infect. Dis..

[50] Fesus A., Benko R., Matuz M., Kungler-Goracz O., Fesus M.A., Bazso T., Csernatony Z., Kardos G. (2021). The Effect of Pharmacist-Led Intervention on Surgical Antibacterial Prophylaxis (SAP) at an Orthopedic Unit. Antibiotics.

[51] Fésüs A., Benkő R., Matuz M., Engi Z., Ruzsa R., Hambalek H.A., Illés Á., Kardos G. (2022). Impact of Guideline Adherence on Outcomes in Patients Hospitalized with Community-Acquired Pneumonia (CAP) in Hungary: A Retrospective Observational Study. Antibiotics.

[52] Charlson M.E., Pompei P., Ales K.L., MacKenzie C.R. (1987). A new method of classifying prognostic comorbidity in longitudinal studies: Development and validation. J. Chronic Dis..

[53] WHO (2023). Collaborating Centre for Drug Statistics Methodology, Definition and General Considerations. https://www.whocc.no/ddd/definition_and_general_considera/.

